# Beyond memories: A narrative review on the role of amygdalo-hippocampal complex in social cognition

**DOI:** 10.1007/s10072-026-09265-0

**Published:** 2026-08-19

**Authors:** Davide Quaranta, Alessandro Scalese, Simona Luzzi, Veronica Cherubini, Annarita Giovagnoli, Stefano Francesco Cappa

**Affiliations:** 1 Department of Neuroscience, Catholic University of the Sacred Heart, Roma, Italy; 2 Department of Psychology, Catholic University of the Sacred Heart, Milano, Italy; 3 Neurology Unit, Fondazione Policlinico Universitario “A. Gemelli” IRCCS, Largo Agostino Gemelli, 1, Roma, Rome 00168 Italy; 4 Department of Experimental and Clinical Medicine, Cognitive and Behavioral Neurology Unit, Polytechnic University of Marche, Ancona, Italy; 5 Department of Diagnostics and Technology, Fondazione IRCCS Istituto Neurologico Carlo Besta, Milan, Italy; 6 Department of Human and Life Sciences, University School for Advanced Studies (IUSS), Pavia, Italy; 7 Department of Neuroscience, IRCCS Istituto Auxologico Italiano, Milan, Italy

**Keywords:** Social cognition, Amygdalo-hippocampal complex, Hippocampus, Amygdala

## Abstract

Social cognition, essential for interpreting and responding to social contexts, is frequently impaired in neurological and psychiatric disorders. Multiple brain regions, organized in large-scale brain networks, represent the neurobiological substrate of social cognitive abilities, and are the main target of neurodegeneration in the fronto-temporal dementia spectrum. The focus of this review is a specific anatomic subcomponent of these networks, i.e. the amygdalo-hippocampal complex (AHC). Animal models suggest that the AHC is involved in processes underlying social cognition through both localized mechanisms and integration within large-scale networks. In humans, AHC pathology in conditions such as Alzheimer’s disease (AD), epilepsy, and autoimmune encephalitis (AIE) has been associated with social cognition impairment. In AD and Mild Cognitive Impairment (MCI) deficits in Theory of Mind (ToM), empathy, and emotion recognition are common and linked to medial temporal atrophy. In focal frontal and temporal lobe epilepsy, seizures and interictal dysfunctions disrupt social cognition, particularly ToM, from early stages. In AIE, marked fear recognition deficits are closely related to amygdala involvement. These conditions highlight the association between social cognition impairments and damage to the AHC. From a theoretical perspective, investigating the relationship between the AHC and social cognition may advance our understanding of the neurobiological underpinnings of social behavior. Clinically, the systematic assessment of social cognition in patients with conditions affecting the AHC appears warranted, potentially improving diagnosis, prognostic evaluation, and therapeutic strategies.

The psychological concept of social cognition and of its neurobiological underpinnings is currently playing a major role in the study of brain disorders. Data from human disease are contributing to the definition of the neural basis of social cognition, which can be broadly defined as a set of abilities that “enable individuals to take advantage of being part of a social group” [1]. According to the framework proposed by Arioli et al. [2], social cognition can be conceptualized as comprising three major domains: social perception, social understanding, and social decision-making. Following a hierarchical subdivision, the first stage of the process is the perception of socially relevant stimuli, mostly in the visual (facial expressions, gestures, postures) but also in the auditory (voice, emotional intonation) domain. This information is integrated and provides input to the mechanisms involved in social understanding, including cognitive and emotional aspects. Understanding of the social context is required for adaptive decision-making about behavioural choices of the organism. One or more steps of this process can be disrupted by neurological or psychiatric disorders. Impairment of social cognition can clinically emerge as a consequence of: (i) poor perception of social cues (i.e. interpreting facial expression, body languages, voices); (ii) reduced emotional empathy (i.e. the emotional response to the perceived situations of others; (iii) abnormal Theory of Mind (ToM, i.e. the ability to understand the mental states of others. Two subtypes can be distinguished: affective ToM, which regards the understanding of others’ emotions, affective states and feelings, and cognitive ToM, which involves the comprehension of cognitive states, beliefs and intentions of others); (iv) social behavior abnormalities (such as poor social tact, lack of manners, inappropriate infringements of interpersonal boundary or failure to reciprocate socially) [3].

At the clinical level, the most important impact of social cognition has come from its contribution to the interpretation of the dysfunctional mechanism responsible for the behavioural manifestations associated with clinical conditions, such as autism [4] and dementia [5]. While there is general consensus about the high prevalence of social cognition deficit in neurological and psychiatric disorders [6], it should be noted that a defective performance in social cognition tasks can be due to impairment of other areas of cognition involved by the task (such as executive functions, visual perception, language or memory).

A debated issue is whether the multiple facets of social cognition share a common factor or represent independent subdomains [7]. At the research level, clinical studies have provided crucial information for social cognitive neuroscience models, which attempt to relate the components of social cognition to their neurobiological substrate [8]. The traditional lesion-based approach as applied to social cognition results in the definition of regionally-based anatomo-clinical correlations of specific components (see, for example, [9]). More recent models are taking into account the contribution of neuroimaging studies in healthy subjects, emphasizing the role of neural networks, rather than of individual brain regions, in supporting different aspects of social cognitive abilities [5, 7]. Several models of brain mechanisms subserving the multiple facets of social cognition have been proposed in the last two decades. The Social Context Network Model (SCNM) underlines the contextual nature of social information processing, proposing a central role of a fronto-insular-temporal network responsible for the updating of contextual cues and their predictive use, the coordination of external and internal milieu and the learning of the value-based association between target and context [10]. The model is strongly grounded in the analysis of the clinical manifestations of the behavioral variant of frontotemporal dementia (Bv-FTD), similarly to the Intrinsically Connected Network model (ICN) [5]. The ICN proposes three brain networks as the pillars of social cognitive processing. The salience network (SN), whose main hubs are the anterior insula and the anterior cingulate cortex, is the key player for the identification of personally salient external or internal stimuli, i.e. the value-associated experiences involved by recognition and engagement with social signals. The second component is the semantic appraisal network (SAN), centered on the anterior temporal lobe, amygdala and orbitofrontal cortex, which is responsible for the precise evaluation of the hedonic value of positively or negatively valenced stimuli or situations, in relation to the self and to others. Finally, the default mode network (DMN), involving the core memory-related structures, including the hippocampal formation (ventral component) and dorsal prefrontal regions, temporo-parietal junction, posterior cingulate cortex and precuneus, is engaged by contextual interpretation of self- and other behavior, providing integrated, high order interpretation of the social signals on the basis of inferences grounded in present and learning-based previous experiences.

Most papers investigating the neural changes leading to impaired social cognition are based on clinico-pathological correlations in bv-FTD, in which the atrophy typically involves brain regions overlapping the SN [11]. On the other hand, several neurological conditions are associated with neuropathological and neurophysiological changes in regions belonging to the SAN and DMN. In this framework, we specifically focused on the role of the amygdalo-hippocampal complex (AHC) in social cognition.

Historically, the hippocampus is considered the main hub of episodic memory, with a major role in encoding, post-encoding consolidation and memory retrieval [12]. The amygdala, on the other hand, is specialized for the processing of emotion and supports hippocampal memory formation facilitating encoding and consolidation of emotionally relevant events [13].

Episodic memory can be expected to impact on empathy, a central aspect of social cognition. The psychological construct of empathy includes multiple processes involved in understanding and sharing the experience of others, including autobiographical memory. For example, in the case of empathy for pain, semantic knowledge is sufficient to recognize the sufferance of another individual. It is, however, possible that the empathic response is required, or at least facilitated, by the recollection of specific episodes of personal painful experiences. Some supporting evidence has been provided by functional magnetic resonance [14] and electroencephalographic [15] studies. Beyond its fundamental role in declarative memory, the contribution of AHC in the context of social cognition deserves appropriate consideration for two main reasons. The first one is that, as described above, according to current views on large-scale brain networks, amygdala is part of both the Salience Network and the Semantic Appraisal Network (basolateral nuclei), while hippocampus is part of the DMN. Amygdala and hippocampus display strong and complex structural connections, thus being among the critical sites for the interaction between the networks. The second reason is that the AHC is the target of neuropathological changes in several neurological diseases in which social cognition may not be routinely assessed in clinical practice with adequate testing materials [16].

This paper, structured as a narrative review, begins with an overview of the functional anatomy of AHC and then focuses on the impact of pathophysiological changes involving the AHC on social cognition, highlighting differences and similarities across different nosological entities. In particular, we will focus on Alzheimer’s disease, epilepsy and autoimmune limbic encephalitis as models of AHC pathology.

The aim of this review is to synthesize evidence supporting the role of AHC as a functionally integrated hub of social cognitive processes. By bridging together evidence from diverse condition, we highlight a consistent association between AHC pathology and deficits in key social cognitive domains. Accordingly, we propose that considering the AHC as a shared neuroanatomical hub for social cognition may provide a useful framework for interpreting these deficits and supports the systematic assessment of social cognition in disorders affecting this region.

## Methods

Relevant literature was identified through searches of major biomedical databases (PubMed and Google Scholar) using combinations of keywords including “amygdala,” “hippocampus,” “amygdalo-hippocampal complex,” “social cognition,” “theory of mind,” “empathy,” “emotion recognition,” “Alzheimer’s disease,” “mild cognitive impairment,” “epilepsy,” and “autoimmune encephalitis.” Priority was given to peer-reviewed original studies, reviews, and seminal articles relevant to the topic. Additional references were identified through citation screening of selected publications. As this work was designed as a narrative review, no formal systematic review protocol or predefined study selection criteria were applied.

### Functional anatomy of the amygdala-hippocampal complex

The amygdala-hippocampal complex is a key structure of the limbic lobe, involved in several functions including memory and emotion appraisal.

In humans and primates, the amygdalar nuclear complex and hippocampal region are juxtaposed in the anterior part of the medial temporal lobe, with the amygdala rostral to the head of the hippocampus.

The hippocampal region includes hippocampal formation and parahippocampal cortices. The former contains the dentate gyrus (DG), the Cornu Ammoni regions (CA1, CA2, CA3), and subiculum; the parahippocampal region includes the perirhinal cortex (PRC), the entorhinal cortex (ERC), the parahippocampal cortex (also known as post rhinal cortex in rat and cat), the presubiculum, and the parasubiculum. The Trisynaptic Circuit [17] is the fundamental pathway through which information flows through the hippocampus. It is formed by the unidirectional connections starting from ERC to the DG to CA3 to CA1. According to classic nomenclature, it includes the perforant pathway (from ERC to DG), mossy fibers (from DG to CA3), and Shaffer’s collaterals (from CA3 to CA1) [18]. The ERC is regarded as the gateway to the hippocampal formation. It receives afferents from the perirhinal and parahippocampal cortices, which are in turn targeted by highly processed inputs from multimodal sensory cortices, mainly involved in vision. Downstream of the trisynaptic circuit, the flow of information continues from CA1 to the subiculum, which projects to the ERC, which projects to the neocortex with a further relay in PRC and parahippocampal cortex.

This somewhat simplified description reflects the classical view of the morpho-functional organization of the hippocampus. More recent evidence has pointed to the presence, for example, of direct connections from the entorhinal cortex to CA3 and CA1, feedback connections from CA3 to DG, and recurrent connections of CA3 neurons on other CA3 neurons [19], subsuming highly complex computational mechanisms.

The amygdala is a complex structure consisting of several nuclei (at least 13 in rodents and 9 in humans) characterized by distinct connections [20]. The nomenclature of amygdalar nuclei varies from one classificatory system to another; in primates they are commonly subdivided into three subgroups: (1) basolateral nuclei, containing the lateral nucleus, the basal nucleus, and the basomedial nucleus; (2) cortical-like groups, which comprises nucleus of the lateral olfactory tract and the cortical nuclei; and (3) centromedial group, encompassing the medial and central nuclei [21]. The basolateral group contains mainly glutamatergic neurons, whilst the centromedial subgroup is mainly formed by GABAergic neurons [22]. The lateral nucleus receives afferent inputs from the sensory thalamus and cortices, and projects to basal and basomedial nuclei. The basolateral complex is reciprocally connected with cortical regions, especially the midline and orbital prefrontal cortices and the hippocampus and displays unidirectional outputs towards the striatum (especially the nucleus accumbens), the bed nucleus of the stria terminalis, and the central nucleus of the amygdala. The latter structures may mediate the translation of basolateral signals to behavioral output.

**Fig. 1:**
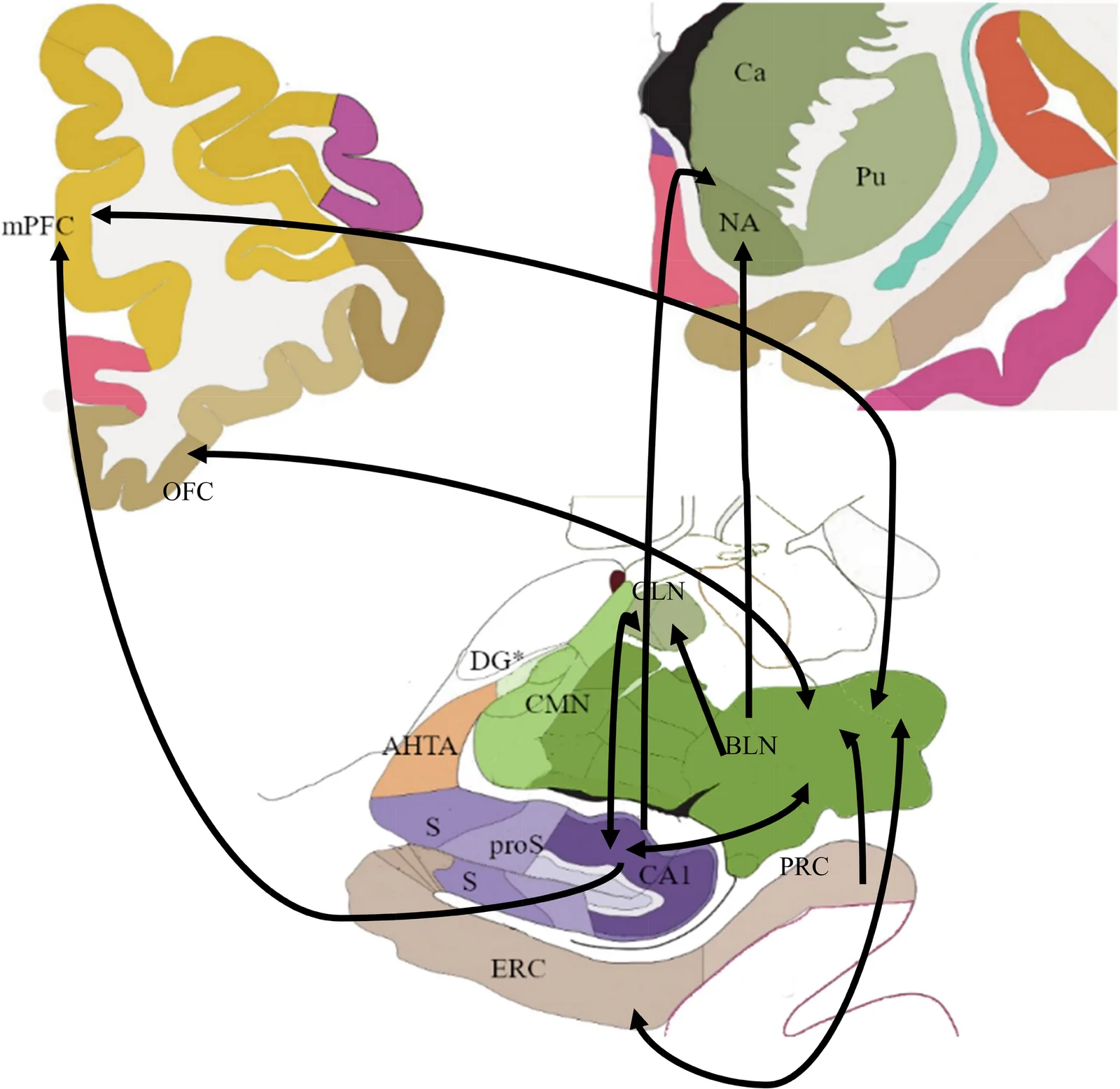
Simplified diagram illustrating the functional and structural connections between the amygdalo-hippocampal complex, cortical regions and basal ganglia relevant to social cognitive processing. Single-headed arrows denote predominantly unidirectional connections, whereas double-headed arrows denote reciprocal (bidirectional) connections. The figure is intended as a conceptual overview of the major pathways discussed in the text and does not represent a comprehensive anatomical map (see the paragraph “Functional anatomy of the amygdala-hippocampal complex” for further explanations). Ca: Caudate Nucleus; Pu: Putamen; NA: Nucleus Accumbens; mPFC: medial Prefrontal Cortex; OFC: Orbitofrontal Cortex; DG: Dentate Gyrus; AHTA: Amygdalo-hippocampal Transition Area; S: Subiculum; ProS: Prosubiculum; CA1: Cornu Ammoni 1; ERC: Entorhinal Cortex; PRC: Perirhinal Cortex; CMN: Centromedial Nuclei; CLN: Cortical-Like Nuclei; BLN: Basolateral Nuclear Complex. Images modified from the Allen’s Institute Human Brain Atlas [75] (https://atlas.brain-map.org/)

The amygdala and hippocampus are strongly connected (see Fig. 1). Projections from the hippocampal formation arise from CA1 and subiculum, and reach the basolateral nuclear complex, cortical nuclei, and anterior portions of the medial nucleus [23]. In rodents, most of these projections arise from the ventral part of subiculum and CA1 (vCA1). This partition corresponds to the anterior part of hippocampus in primates, which is involved in affect and emotion [24] and, besides to the amygdala, projects to medial prefrontal cortex (mPFC) and nucleus accumbens. ERC, PRC and parahippocampal cortex have complex connections with amygdala. The main projections from ERC to amygdala arise from the lateral part of ERC, targeting the basolateral complex. The perirhinal cortex also projects to the basolateral complex, central nucleus, and cortical nuclei. The parahippocampal cortex has a projection to the lateral nucleus [23].

With few exceptions, the projections from amygdala to hippocampus reciprocate the projections from hippocampus. In particular, ventral subiculum and ventral CA1 receive projections from the basolateral nucleus complex [21] and ERC receives inputs from the lateral, posterior basolateral, basomedial, medial, posterolateral cortical, and posteromedial cortical nuclei [25].

The role of the amygdalo-hippocampal complex in social abilities has been emerging in recent years with contributions from animal and human research. In particular, the basolateral complex seems to exert a significant role, since its inhibition favours social behavior, whereas its activation reduces social behavior [26]. Similar effects were also observed when the connections between basolateral complex and vCA1 were manipulated [27]. On the other hand, vCA1 has been associated with social memory, that is the ability to recognize familiar conspecifics [28]; the interaction between vCA1 and dorsal CA2 may be particularly relevant for this specific aspect of social behavior [29]. CA2 has been proposed to play a central role in social memory, the basis of the multiple aspects of social recognition, from familiarity to social hierarchy [30].

The basolateral-hippocampus interaction may be also relevant in some aspects of social exploration [31]. Bickart et al. [32] reported a direct correlation between the amygdala volume and the size (measured as number of regular contacts) and complexity (measured as number of embedded groups) of social networks. More in general, the connections between basolateral amygdala and hippocampus could be a site of interaction between SAN and DMN [5].

It is important to note that, given the central role of the AHC in memory formation, long-term memory processes and empathic abilities are difficult to fully disentangle. In fact, social interactions rely at least in part on the capacity to retrieve and integrate autobiographical and semantic memories about one’s own and others’ past experiences, in order to simulate or anticipate others’ emotional and mental states [33, 34]. Episodic memory supports the construction of rich, context-specific representations of prior social encounters, whereas semantic memory provides more abstract knowledge about social norms, roles, and typical emotional reactions [35]. Together, these memory systems enable individuals to generate informed predictions about how others are likely to feel or behave in a given context [36].

Taken together, these considerations suggest that in neurological diseases classically characterized by memory impairment, alterations in social cognition may, at least in part, reflect downstream consequences of the underlying memory dysfunction. Conversely, a “pure” deficit in social cognition should only be inferred when the integrity of long-term memory systems has been clearly established. From another and somehow opposite perspective, according to the ICN model, the AHC may exert an integrative role by supporting the interaction between SAN and SN and thus contributing to the occurrence of an impairment in social cognition that extends beyond the effects of episodic memory disturbances.

A schematic overview of the principal domains of social cognition and their putative association with the AHC are presented in Table 1.

**Table 1:** Overview of the three major domains of social cognition, according to Arioli et al. [2], together with the putative role of the AHC as suggested by the literature reviewed in this article

Social-cognitive domain	Representative functions	Putative role of the AHC
Social Perception	Emotion recognition, processing of socially relevant stimuli	Amygdala-mediated salience detection and emotional appraisal; interaction with hippocampal contextual representations
Social Understanding	Theory of Mind, empathy	Integration of emotional relevance (amygdala) and contextual/autobiographical information (hippocampus)
Social Decision-Making	Social judgments, moral reasoning, behavioral adaptation in social contexts	Integration of prior experiences, emotional value, and contextual information through interactions between SAN and DMN

*AHC* Amygdalo-hippocampal complex, *SAN* Semantic appraisal network, *DMN* Default mode network

### Alzheimer’s disease and mild cognitive impairment

Temporo-mesial atrophy is a hallmark of AD, in particular of the most frequent variant of AD, i.e. the amnestic subtype [37]. Moreover, a body of evidence from recent imaging studies has suggested that the amygdala might play a major role in AD, even in early stages of disease and especially regarding neuropsychiatric symptoms [38]. Thus, AD can provide a valuable neuropathological model to assess the role of AHC on social cognition. Furthermore, the assessment of social cognition in Mild Cognitive Impairment, which is prodromal to dementia, offers the possibility to explore the status of social cognition in the early phase of the disease, when the neuropathological changes are relatively limited.

The nature of impaired social cognition in AD is a matter of debate. Historically speaking, in contrast to frontotemporal dementia, social cognition impairment in AD has been less extensively studied, possibly because it is usually overshadowed on standard clinical and neuropsychological evaluation by the prevalent amnesic syndrome. However, nowadays, substantial evidence demonstrated that AD patients may also show impairment of emotion perception, ToM and loss of empathy.

Overall, the literature on social cognition in AD and MCI, although vast, is characterized by a high variability of the results. When compared to healthy controls, MCI and AD patients display a similar profile characterized by impaired performance in tasks assessing, for example, second-order beliefs (“I am inferring the mental state of a person about another person”) or in tasks such as the Faux Pas Recognition [39, 40]. When subcomponents of social cognition (e.g., “cognitive” vs. “affective” dimensions of empathy and/or Theory of Mind, socio-emotional processing, emotion recognition) have been explored conflicting results emerged. For example, the majority of studies has provided evidence for impairment on ToM components in MCI, although with high heterogeneity in tests used and domain tested [41]; however, others found no significant impairment in MCI patients [42]. Overall, ToM deficits were proved to be more severe in AD than in amnestic MCI (aMCI) in most individual tasks [43]. Similarly, in Facial Emotion recognition (FER) discordant results are present, depending on the severity of disease and the heterogeneity of the used methods [44].

In this context, some studies on AD patients have pointed out the possible roles of the AHC. One paper demonstrated that amygdala is important for sustaining and regulating emotions independently from both hippocampal volume and declarative memory [45]. Further insight about social abilities has been reported to be associated with AHC atrophy in a study involving patients with different neurodegenerative disorders (including AD) [46]; in particular, atrophy of left amygdala and hippocampus has been associated with reduced insight in emotion recognition, whereas right amygdala atrophy was associated with reduced insight on social interactions.

To the best of our knowledge, only three studies evidenced correlations between specific social tasks and hippocampus. One study found that TASIT performance correlated with hippocampal volume bilaterally together with the right amygdala, bilateral accumbens and bilateral putamen. They also found that performance on the Ekman 60 was also significantly associated with the right hippocampus volume [47]. Others found that learning and memory of social interactions was associated with medial temporal and temporoparietal atrophy in AD [48]. In Synn et al. the right hippocampus volume correlated with mentalizing capacity performance in AD [49].

Valuable insight about this topic can be drawn by studies about behavioral variant AD (bvAD). Compared with bvFTD, bvAD patients had less involvement of the SN and SAN, showing a more extensive overlap of neuroimaging features with typical amnestic-predominant AD (tAD) and supporting the idea that bvAD is a different nosological entity from bvFTD with AD co-pathology. Regarding the AHC, bvAD showed more anterior DMN involvement and a larger amygdalar volume, without a significant difference in hippocampus grey matter volume, in comparison with tAD. Furthermore, amygdalar volume was found to be the best radiological discriminating factor between bvAD and tAD, allowing to speculate about the early relative sparing of amygdala from AD pathology and its potential role in shaping the clinical phenotype of bvAD. From a clinical point of view, bvAD patients were found to have a more severe social impairment and divergent eye movement patterns compared to tAD [50]. Resting state connectivity studies in MCI patients documented a stronger inter-network connectivity between the insular hub of the SN and the executive fronto-parietal network, in a context of dysfunctional DMN due to early AD neurodegeneration. This modulation might reflect an adaptive response of the executive network, expressed in neural territories relatively spared by AD pathology in prodromal stages, thus providing support sustaining social cognition [51]. This and other putative adaptive mechanisms might explain why some studies found little evidence of social cognition impairment in the MCI population. A study based on functional MRI (fMRI) showed that amnestic MCI subgroup, compared to non-amnestic MCI subgroup, had worse ToM performances and reduced functional connectivity between the bilateral temporal lobe and the left lateral temporal cortex [52].

### Epilepsy

Fisher et al. [53] defined epilepsy as “a disease of the brain characterized by an enduring predisposition to generate seizures and the neurobiological, cognitive, psychological, and social consequences of this condition.” Therefore, not only disease-related factors (e.g., brain injury, epileptic discharges, antiepileptic drugs, AED), but also sociodemographic variables can cause cognitive impairment. This is especially true for social cognition, which may reflect neurobiological and psychosocial aspects, such as integration, employment status, and cultural level.

Focal epilepsies that originate in the frontal and temporal lobes are the most common types of epilepsy and also those that most frequently result in impaired social cognition, due to impact on key areas and pathways of its underlying circuits. Epileptic discharges that begin within the frontal lobes tend to propagate to other brain areas immediately and at high speed, so patients with frontal lobe epilepsy (FLE) may have varying degrees of impairment of social cognition components, particularly ToM. The experimental results are however conflicting. Farrant et al. [54] found deficits in executive tasks, but normal performance in a faux pas test requiring the comprehension of epistemic and affective mental states. The patients, however, were not accurate in recognizing facial emotions (anger, fear, and sadness) and eye-expressed mental states. A subsequent study showed significant impairment in faux pas recognition in patients with FLE compared with healthy controls and patients with temporal lobe epilepsy (TLE), depending also on the duration of epilepsy [55]. In TLE, discharges often originate in regions of the medial temporal lobe, involving major components of the ToM network, such as the amygdala, hippocampus, superior temporal sulcus and temporoparietal junction, and spread to the limbic system and frontal lobes. Initial studies revealed that adult patients with medial TLE may show selective deficits of ToM resulting from congenital or early amygdala damage. In the aforementioned study conducted on 138 nonsurgical patients, Giovagnoli et al. showed that TLE can significantly impair ToM. As patients with FLE, those with TLE were able to exclude nonexistent faux pas. The impairment of verbal comprehension and fluency, abstract reasoning, visuospatial perception, seizure frequency, and AEDs did not predict ToM deficits, while early age of seizure onset, along with medial temporal lobe sclerosis, were significantly associated with ToM impairment [55]. In 21 patients with TLE, Li et al. [56] suggested that TLE can affect not only advanced but also basic ToM skills, a finding confirmed by Wang et al. using tests of False Belief, Faux Pas Recognition, Implication Stories, and Visual Cartoon [57]. The latter also showed no significant differences between patients with right and left TLE and reported the worst ToM performance in patients with bilateral TLE, suggesting the absence of brain lateralization for ToM. However, recent studies [58] have also reported appropriate levels of empathy in patients with TLE, alongside mild impairment in moral reasoning and deficits in recognition of social situations. One year after anterior temporal lobectomy for drug-resistant TLE, ToM was found to be unchanged or slightly improved depending on cognitive reserve and age of seizure onset [59]. Together with the findings on the lack of effect of AEDs, this suggests that surgical and medical treatment of seizures does not substantially change ToM.

In summary, the study of social cognition in epilepsy began long after studies on memory and language, highlighting an initial lack of attention to the cognitive-behavioral sphere in this condition, despite its clinical relevance [53]. Almost all findings demonstrated that ToM may be impaired in focal epilepsies involving the frontal and medial temporal lobe areas in patients with early seizure onset. Epileptic discharges in the absence of detected brain damage may influence ToM abilities, explaining the presence of deficits in cryptogenic and generalized epilepsies. Processing of social situations may also be affected in TLE, while empathy and moral reasoning are preserved.

### Autoimmune encephalitis

Autoimmune encephalitis (AIE) is a neurological disorder that develops as a rapidly progressive encephalopathy caused by brain immune-mediated inflammation. A prominent involvement of AHC is found in autoimmune limbic encephalitis (ALE) [60]. Inflammatory processes of mesiotemporal structures can lead to functional disturbances and potentially to irreversible chronic damage [61]. ALE is clinically characterized by a subacute onset (less than three months) of working memory deficit, seizures or psychiatric symptoms, associated with bilateral brain abnormalities on T2-weighted fluid-attenuated inversion recovery MRI restricted to the medial temporal lobe. In recent years, several auto-antibodies against surface antigens (e.g., leucine-rich glioma inactivated protein 1 (LGI1), contactin associated protein-like 2 (CASPR2)), likely directly pathogenic, were discovered [62]. LGI1 encephalitis is the most common type of ALE, usually presenting in middle-aged men and having faciobrachial dystonic seizures as unique feature. Anti-LGI1 antibodies seem to exert their action by several mechanisms, which imply the disruption of synaptic protein complexes (LGI1 is a trans-synaptic protein), AMPA receptor internalization with reduced synaptic density, leading to neuronal hyperexcitability. The typical cognitive impairment of LGI1 encephalitis is characterized by autobiographical episodic memory deficit, disorientation and global confusion. CASPR2 encephalitis, while associated with a wide range of neurological syndrome, including peripheral nerve excitability or Morvan syndrome, may present as limbic encephalitis. Encephalitis associated with antibodies against glutamate-specific N-Methyl-d-aspartate receptor (NMDAR), although not canonically classified as limbic encephalitis, may frequently present with prominent deficits in episodic memory and executive functions, due to the high concentrations of NMDARs in the hippocampus and frontal cortex [63].

Whereas many studies support the presence of episodic memory deficit in ALE, the present literature about social cognition in this nosological entity is limited [64, 65]. Emotion recognition is the most explored field. The largest study available based on 69 immunotherapy-naïve ALE-patients, found deficit in emotion recognition with most pronounced difficulties in recognizing fear [66]. Furthermore, reduced arousal ratings and autonomic responsivity to anxiety inducing stimuli were found in patients with ALE [67, 68]. In particular, the degree of right amygdala enlargement predicted the autonomic response to fear-inducing conditions, suggesting functional lateralization of the right amygdala and emphasizing its role as a primary basis of emotion-related physiological responses. Significant deficits of vocal and facial expression recognition of disgust and fear were also found in a patient with paraneoplastic limbic encephalitis associated with anti-Ma2 antibodies [69]. To our knowledge, only one paper studied other aspects of social cognition in LGI1 or CASPR2 ALE patients: no cognitive ToM deficit measured by Story-based Empathy Task-SET, was found, as well as no impairment of the processing of emotional cues, investigated with the Reading the Mind in the Eye Test – RMET. However, this case series included only three patients [70]. In one study, anti-NMDAR encephalitis patients performed worse than controls in tasks assessing the severity of interpersonal violations and the use of mental state information to understand social situations. Emotion recognition and the ability to recognize conventional/unconventional social behaviours were spared [71].

Overall, ALE patients show a specific vulnerability to the fear recognition. Lots of evidence support the fact that fear is more dependent on the amygdala than other emotions. Its impairment disrupts fear processing more than other emotional systems that may rely on broader and more redundant brain networks.

Further research is needed to assess social cognition in ALE patients. A deeper understanding of the relationship between inflammation, antibodies and social cognition impairment may enhance diagnostic and prognostic accuracy, emphasizing the need for early treatment initiation in patients with ALE.

## Conclusions

This narrative review examined the contribution of AHC to social cognition by integrating evidence from anatomical studies and from neurological disorders characterized by prominent involvement of medial temporal structures. Although the hippocampus and amygdala have traditionally been investigated in relation to episodic memory and emotional processing, respectively, the findings reviewed here support that their functional relevance extends to multiple domains of social cognition.

Evidence from AD, TLE and ALE consistently demonstrates that pathological processes affecting the AHC are frequently associated with deficits in emotion recognition, ToM, empathy, social judgment, and interpretation of social situations. Importantly, these deficits emerge despite substantial differences in etiology, disease course, and underlying pathophysiological mechanisms. Such convergence supports the hypothesis that the AHC represents a shared neuroanatomical substrate contributing to social cognitive functioning across different neurological conditions.

Current network-based models provide a useful framework for interpreting these observations. Within the ICN model, the amygdala participates in the SN and SAN, whereas the hippocampus represents a key component of the DMN. The extensive reciprocal connectivity between these structures places the AHC in a strategic position for integrating emotional relevance, contextual information, prior experience, and self-referential processing. Consequently, dysfunction of the AHC may disrupt communication between networks that are essential for understanding and predicting the behavior of others. However, to our knowledge, no human studies have directly examined whether amygdala–hippocampal connectivity specifically contributes to social cognition beyond the contribution of either structure considered separately, whereas preliminary evidence is available from animal models [27].

An important consideration (and potential limitation) is that the evidence reviewed herein does not establish a direct causal role of AHC in all aspects of social cognition. The clinical conditions discussed are characterized by broader network dysfunction extending beyond the AHC and frequently involve impairments in executive functions, attention, language and memory (as discussed above, memory dysfunction, a common consequence of AHC pathology, is highly relevant to this issue, as social interactions often rely on the retrieval and integration of prior social experiences and stored social knowledge). Consequently, the observed associations between AHC pathology and social cognitive deficits should be interpreted with caution, as they may reflect both direct effects of AHC dysfunction and indirect consequences of disturbances in distributed neural networks, whose respective contributions remain difficult to disentangle. Furthermore, social cognition relies on the integration of multiple cognitive processes, including memory, contextual representation, emotional processing, and executive control. In this framework, deficits in theory of mind, empathy, or emotion recognition may emerge not only from impairment of social-cognitive mechanisms per se but also from disruption of “upstream” cognitive functions that support social behavior. For example, conflicting evidence has been provided by the investigation of patients with hippocampal amnesia. Two cases due to severe traumatic brain injury were studied by Rosenbaum et al. [72]. Notwithstanding the severe loss of episodic memory, their performance on an extensive assessment of ToM tests was faultless, suggesting an independence of the two functions in adult life. On the other hand, Beadle et al. studied specifically empathy in three patients with post hypoxic amnesia, and results showed lower trait empathy than the controls, as well as no response to empathy induction [73]. Future studies combining detailed neuropsychological assessment with network-based neuroimaging approaches will be essential to disentangle the specific contribution of the AHC from that of broader cognitive and neural systems. According to the ICN model, the AHC may directly contribute to social cognitive processes through the integration of emotional salience, contextual information, and mental-state representations [5]. As an example, a recent study combining computational modeling and fMRI demonstrated that the medial temporal lobe is involved in the abstract cognitive representation of the numerous social ties linking individuals within a community [74].

Other limitations should be acknowledged. First, the available literature is highly heterogeneous with respect to clinical populations, disease severity, social cognition measures and neuroimaging methodologies. Second, most studies investigated individual components of social cognition rather than providing comprehensive assessments across multiple domains. Finally, relatively few studies specifically examined the contribution of the AHC as an integrated functional unit, with most focusing on either the amygdala or hippocampus in isolation. Furthermore, the current lack of evidence concerning the specific contribution of amygdala–hippocampal connectivity to social cognition precludes definitive conclusions about whether these structures operate as a unitary computational system. Preliminary evidence from animal models nevertheless suggests that some aspects of social behavior depend on bidirectional interactions between the amygdala and hippocampus [27]. These findings have not yet been replicated in humans. Therefore, rather than representing an evidence-established conclusion, the proposed computational role of the AHC in social cognition should currently be regarded as a conceptual framework (Fig. 1).

Future investigations should employ standardized social cognition batteries, multimodal neuroimaging approaches, and longitudinal designs to better characterize the evolution of social cognitive deficits in disorders affecting the medial temporal lobe. Moreover, clarifying the mechanisms linking AHC pathology to social dysfunction may assist clinicians in identifying specific neuropsychological profiles associated with particular etiologies, enhancing diagnostic accuracy and potentially aiding clinicians in the management of these conditions with cognitive rehabilitation and, when available, with pharmacological interventions.

In conclusion, the available evidence supports a broader conceptualization of AHC as a critical hub for social cognition. Beyond its established role in memory and emotional processing, the AHC appears to contribute to the integration of contextual, affective, and autobiographical information required for adaptive social behavior. Recognizing this role may improve our understanding of social cognitive impairment across neurological diseases and encourage the routine assessment of social cognition in conditions involving medial temporal lobe pathology.
